# Viral vector vaccines expressing nucleoprotein and phosphoprotein genes of avian bornaviruses ameliorate homologous challenge infections in cockatiels and common canaries

**DOI:** 10.1038/srep36840

**Published:** 2016-11-10

**Authors:** Marita Olbert, Angela Römer-Oberdörfer, Christiane Herden, Sara Malberg, Solveig Runge, Peter Staeheli, Dennis Rubbenstroth

**Affiliations:** 1 Institute for Virology, Medical Center – University of Freiburg, Faculty of Medicine, University of Freiburg, Hermann-Herder-Str. 11, D-79104 Freiburg, Germany; 2 Institute of Molecular Virology and Cell Biology, Friedrich-Loeffler-Institut (FLI), Federal Research Institute for Animal Health, Südufer 10, D-17493 Greifswald – Insel Riems, Germany; 3 Institute for Veterinary Pathology, University Justus Liebig Gießen, Frankfurter Str. 96, D-35392 Gießen, Germany

## Abstract

Avian bornaviruses are causative agents of proventricular dilatation disease (PDD), an often fatal disease of parrots and related species (order Psittaciformes) which is widely distributed in captive psittacine populations and may affect endangered species. Here, we established a vaccination strategy employing two different well described viral vectors, namely recombinant Newcastle disease virus (NDV) and modified vaccinia virus Ankara (MVA) that were engineered to express the phosphoprotein and nucleoprotein genes of two avian bornaviruses, parrot bornavirus 4 (PaBV-4) and canary bornavirus 2 (CnBV-2). When combined in a heterologous prime/boost vaccination regime, NDV and MVA vaccine viruses established self-limiting infections and induced a bornavirus-specific humoral immune response in cockatiels (*Nymphicus hollandicus*) and common canaries (*Serinus canaria* forma domestica). After challenge infection with a homologous bornavirus, shedding of bornavirus RNA and viral loads in tissue samples were significantly reduced in immunized birds, indicating that vaccination markedly delayed the course of infection. However, cockatiels still developed signs of PDD if the vaccine failed to prevent viral persistence. Our work demonstrates that avian bornavirus infections can be repressed by vaccine-induced immunity. It represents a first crucial step towards a protective vaccination strategy to combat PDD in psittacine birds.

Avian bornaviruses are members of the family *Bornaviridae*, which are enveloped viruses with a non-segmented negative strand RNA genome. In cell culture and infected individuals, they establish persistent infections without inducing a cytopathic effect^1^,^2^. Genetically distinct bornaviruses were identified in a broad range of avian species, including Psittaciformes, Passeriformes, Anseriformes and Charadriiformes^3^,^4^,^5^,^6^,^7^,^8^,^9^,^10^,^11^.

At least eight different psittacine bornaviruses (parrot bornavirus 1 to 8; PaBV-1 to 8) are present in captive populations of psittacine birds worldwide^1^,^10^,^12^. Among these psittacine viruses, PaBV-2 and PaBV-4 are most widely distributed^10^,^13^ and both were experimentally confirmed to be causative agents of proventricular dilatation disease (PDD)^14^,^15^,^16^,^17^,^18^,^19^.

PDD in psittacines was first described in the late 1970s. Typical microscopic lesions are mononuclear infiltrations in the central nervous system and peripheral ganglia in various mainly gastrointestinal organs. As a consequence, neurological or gastro-intestinal symptoms are the dominating clinical signs of PDD. Neurological signs include apathy, lameness, ataxia, torticollis, tremor, seizures and blindness. Gastro-intestinal disease is caused by inflammatory lesions of the vegetative nervous system which result in reduced intestinal motility, impaired transport of the ingesta, dilated proventriculus and crop, shedding of undigested seeds with the faeces and emaciation. The course of disease may vary from sudden death without previously expressed symptoms to life-long chronic disease. Complete recovery is rarely reported (reviewed in ref. 20). However, many persistently infected psittacines will remain free of clinical disease for months or even throughout their lives^15^,^17^,^19^,^21^,^22^.

To date, a causative therapy of PDD or a specific immunoprophylaxis against avian bornavirus infections are not available despite their worldwide distribution and the high impact of PDD on psittacines, including captive populations of endangered species such as the Spix's macaw (*Cyanopsitta spixii*) and blue-throated macaw (*Ara glaucogularis*)^23^. Interferon-α and Ribavirin were shown to efficiently inhibit avian bornaviruses in cell culture, but confirmation *in vivo* is lacking^24^,^25^,^26^. Thus, the aim of this study was to develop and evaluate vaccines for the protection against avian bornavirus infections and subsequent disease.

Information on the protective immune mechanisms against bornaviruses in birds is scarce. Viral persistence in the presence of high levels of bornavirus-specific antibodies suggests only a minor role of humoral immunity^5^,^15^,^17^,^21^,^22^. This is in agreement with experimental work performed in rodents with the related mammalian Borna disease virus 1 (BoDV-1) which demonstrated CD4^+^ and CD8^+^ T lymphocytes rather than antibodies to be responsible for the protection against *de novo* infection^27^,^28^,^29^,^30^,^31^. However, CD4^+^ and CD8^+^ T lymphocytes were also demonstrated to drive the immunopathogenesis of Borna disease (BD)^29^,^32^,^33^,^34^,^35^, an immune-mediated chronic neurologic disorder occurring in a wide range of naturally and experimentally BoDV-1-infected mammals, such as horses, sheep, rats and mice (reviewed in refs 2 and 36). BD is characterized by microscopic lesions closely resembling those observed in the central nervous system of PDD-affected psittacines^2^,^20^. Based on the close relationship of BoDV-1 and avian bornaviruses, the similarity of microscopic lesions typical for BD and PDD in the CNS, we hypothesized that PDD is likewise an immune-mediated disease in which bornavirus-specific T lymphocytes can be decisive for both, immunopathology and protection. We further hypothesized that a vaccination regime mounting a strong specific T lymphocyte response is necessary for achieving an early elimination of incoming challenge virus and thereby preventing adverse effects of T cell-mediated immunopathology.

For efficient stimulation of a strong T lymphocyte response including cytotoxic T lymphocytes, active vaccine-induced protein synthesis in the host cells is required^37^. Recombinant viral vector vaccines offer this feature in addition to several further advantages, including well-characterized safety profiles, efficient replication in cultivation systems and potent induction of a broad range of host immune mechanisms^37^,^38^,^39^. The lentogenic Newcastle disease virus (NDV) Clone 30 and the heavily attenuated poxvirus strain modified vacciniavirus Ankara (MVA) are well-established vector platforms^40^,^41^. NDV Clone 30 is derived from a commercial NDV live vaccine which is widely used in poultry flocks^40^. MVA was adapted to chicken embryo fibroblast (CEF) cultures by more than 570 cell culture passages during which several deletions of large genomic regions occurred. While still able to infect a broad range of host cells and to induce protein synthesis, MVA infection is abortive in most cell types other than CEF^38^,^42^,^43^. Experimental studies have demonstrated that recombinant MVA vaccines are safe and efficient not only in humans and other mammals, but also in chicken^44^,^45^. Both, MVA and NDV, were demonstrated to induce a specific cell-mediated immune response in mammals and/or chicken^39^,^44^,^46^,^47^.

In this study we designed recombinant MVA and NDV that encode the nucleoprotein (N) and phosphoprotein (P) genes of two avian bornaviruses, namely PaBV-4 and canary bornavirus 2 (CnBV-2). Bornavirus N and P proteins are strongly expressed in bornavirus-infected cells^48^ and were demonstrated to be immunogenic in BoDV-1 infection models in rodents^28^,^49^,^50^. The newly generated vaccines were used for vaccination of psittacines (cockatiels, *Nymphicus hollandicus*) or non-psittacine birds (common canaries, *Serinus canaria* forma domestica) in a heterologous prime/boost regime and safety, immunogenicity and protection against homologous bornavirus challenge were investigated in both species.

## Results

### Recombinant NDV and MVA vaccine viruses stably express bornavirus N and P proteins in cell culture

In this study, a set of recombinant NDV vector vaccines was generated to express the N or P protein genes of PaBV-4 (designated rNDV/PaBV-4/N and rNDV/PaBV-4/P) and CnBV-2 (designated rNDV/CnBV-2/N and rNDV/CnBV-2/P). Furthermore, a similar set of vaccine viruses was designed using the MVA vector platform (designated rMVA/PaBV-4/N, rMVA/PaBV-4/P, rMVA/CnBV-2/N, and rMVA/CnBV-2/P). All constructs were confirmed to express the respective bornavirus antigen in infected CEF cultures by immunofluorescence staining (see Supplementary Fig. S1). The genetic stability of the recombinant NDV (rNDV) and MVA (rMVA) constructs was confirmed by bornavirus antigen expression following propagation for five passages in either embryonated chicken eggs or CEF cultures, respectively (see Supplementary Fig. S2).

### Vaccination with NDV and MVA vaccine viruses is safe in cockatiels

**Figure 1:**
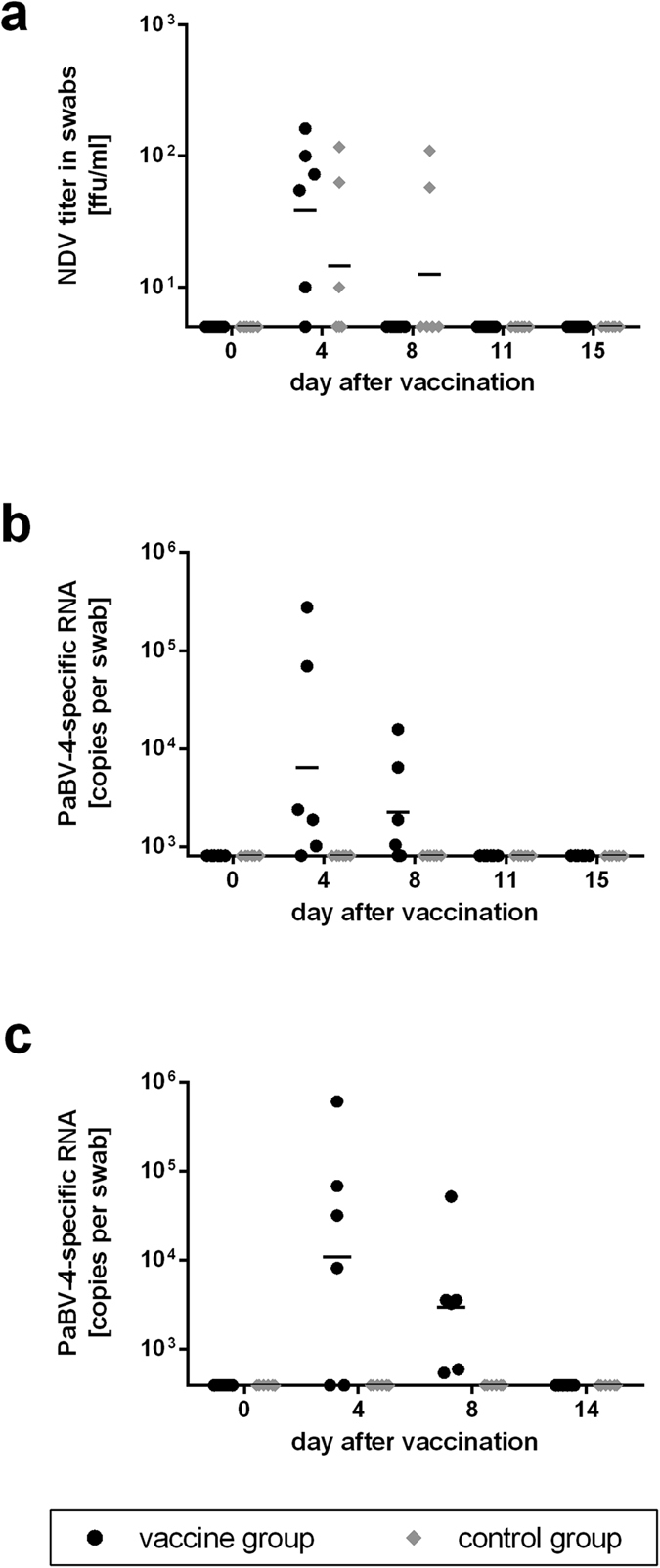
Shedding of NDV vaccine viruses after vaccination of cockatiels (experiments 1 & 2). During two experiments, groups of six cockatiels were either vaccinated with a mixture of rNDV/PaBV-4/N and rNDV/PaBV-4/P vectors (vaccine groups) or received rNDV-wt (control group) and combined pharyngeal and cloacal swabs were collected at the indicated time points after vaccination. (**a**) In experiment 1, infectious NDV was detected by virus titration. (**b,c**) Vector-derived PaBV-4 P RNA originating from rNDV/PaBV-4/P in experiment 1 (**b**) or experiment 2 (**c**) was quantified by RT-qPCR. Positions of the X axes indicate the detection limits of the respective tests.

In two vaccination experiments (experiments 1 and 2), groups of six cockatiels each were vaccinated with either mixtures of rNDV/PaBV-4/N and rNDV/PaBV-4/P (vaccine groups) or with the recombinant NDV vaccine strain “clone 30” not carrying a foreign gene (designated rNDV-wt; control groups). At day 21 after vaccination they were booster-vaccinated with mixtures of rMVA/PaBV-4/N and rMVA/PaBV-4/P or the parental strain MVA-F6 (designated MVA-wt), respectively. All vaccines were administered by intramuscular injection. NDV detection from combined pharyngeal and cloacal swabs confirmed that the virus replicated in cockatiels and reached the mucosal surfaces. Low levels of infectious virus were detected in both groups of experiment 1 at days 4 and 7 after vaccination (Fig. 1a), while NDV-specific RNA was detectable up to day 11 after vaccination (see Supplementary Fig. S3). PaBV-4 P RNA originating from the rNDV/PaBV-4/P vaccine was detected by RT-qPCR in the vaccine groups but not in the control groups (Fig. 1b,c). This finding confirms that NDV vaccine viruses were not transmitted to the control groups which were housed in a separate aviary within the same room. In experiment 1 neither infectious MVA nor vaccine-derived PaBV-4 P DNA was detectable in swabs collected after MVA booster vaccination (data not shown), while in experiment 2 very low copy numbers of MVA-derived PaBV-4 P DNA were detected in a small number of swabs collected from the vaccine group between days 7 and 21 after booster vaccination (equalling days 21 to 35 after the first vaccination; see Supplementary Fig. S3).

None of the animals showed clinical signs following vaccination with the various vaccine viruses. However, one bird (animal A3) of the vaccine group of experiment 1 died during blood sampling at day 21 after vaccination with NDV. The bird had not shown clinical symptoms prior to death and neither macroscopic nor microscopic tissue lesions were observed, suggesting that death was related to sampling-induced stress.

### Heterologous prime/boost vaccination with NDV and MVA vaccines viruses induces a humoral immune response in cockatiels

**Figure 2:**
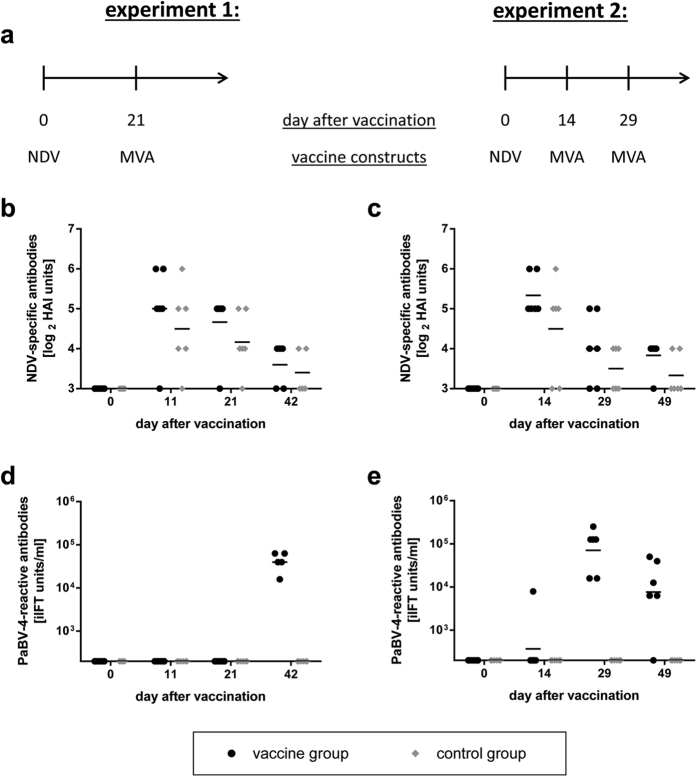
Induction of NDV- and PaBV-4-reactive antibodies after vaccination of cockatiels with NDV and MVA vector vaccines (experiments 1 & 2). (**a**) In two experiments, groups of six cockatiels (vaccine group) were vaccinated with mixtures of vectors rNDV/PaBV-4/N and rNDV/PaBV-4/P. At day 21 after vaccination (experiment 1) or at days 14 and 29 after vaccination (experiment 2), the birds received booster vaccinations with mixtures of rMVA/PaBV-4/N and rMVA/PaBV-4/P. At the same time points, a second group of six birds (control group) in each experiment received the respective parental vaccine strains rNDV-wt and MVA-wt. Plasma samples were collected at the indicated time points and tested for the presence of NDV-specific antibodies by HAI test (**b,c**) or for the presence of PaBV-4-reactive antibodies by iIFT (**d,e**). The positions of the X axes indicate the detection limit of the respective test.

In both cockatiel experiments NDV-specific hemagglutination-inhibiting (HAI) antibodies were detectable already at day 11 or 14 after vaccination with no apparent differences between the groups. Thereafter, HAI titres gradually decreased (Fig. 2b,c). In response to vaccination with NDV vaccine viruses, PaBV-4-reactive antibodies were detectable only in one bird of the vaccine group of experiment 2 (Fig. 2d,e). However, two to three weeks after booster-vaccination with MVA constructs (equalling 28 to 42 days after priming with NDV), all birds of the vaccine groups had developed PaBV-4-specific antibodies, while the birds of the control groups remained negative (Fig. 2d,e). Surprisingly, a second booster vaccination with MVA vaccines performed two weeks after the first booster vaccination in experiment 2 did not appear to result in an increased humoral response but antibody titres had rather decreased three weeks thereafter (equalling day 49 after priming with NDV constructs; Fig. 2e).

### The course of homologous PaBV-4 challenge infection in cockatiels is delayed by heterologous prime/boost vaccination with NDV and MVA vaccines expressing PaBV-4 N and P genes

To evaluate the protective effect provided by the vaccination regime, a homologous challenge infection with isolate PaBV-4 #6758 was performed in experiment 1. Six weeks after the first vaccination with NDV vaccine viruses and three weeks after booster vaccination with MVA constructs, birds of both groups were inoculated with 10^4.6^ foci-forming units (ffu) PaBV-4 per bird by combined peroral, oculonasal, subcutaneous and intramuscular route.

**Figure 3:**
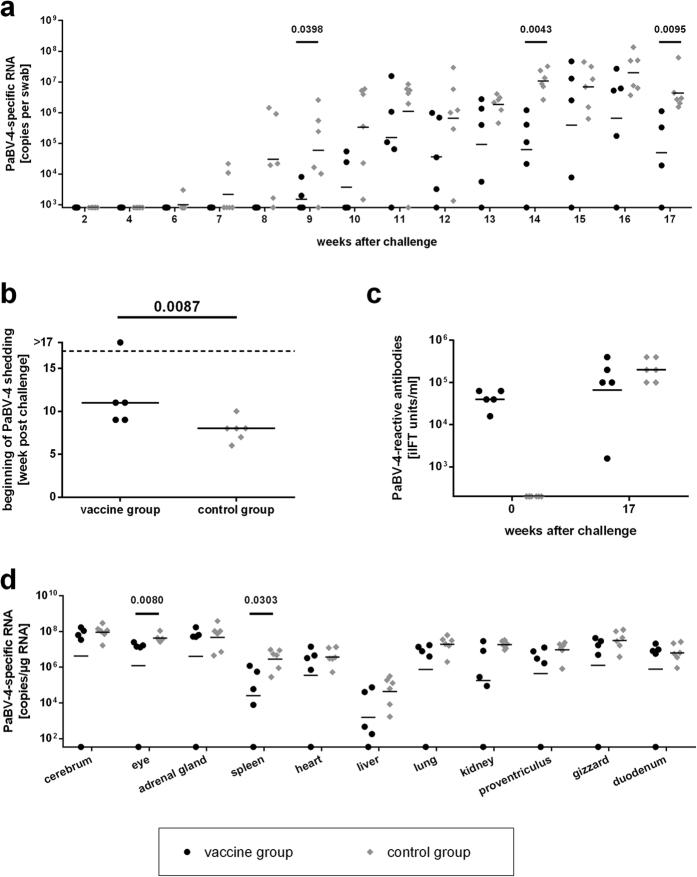
Heterologous prime/boost vaccination of cockatiels against PaBV-4 delays the course of PaBV-4 challenge infection (experiment 1). Two groups of five or six cockatiels had been either vaccinated with a mixture of rNDV/PaBV-4/N and rNDV/PaBV-4/P and boosted with rMVA/PaBV-4/N and rMVA/PaBV-4/P (vaccine group) or received the respective parenteral vaccine strains (control group). Three weeks after booster vaccination, both groups received a homologous challenge infection with PaBV-4. (**a,b**) Cloacal swabs were collected at intervals of one to two weeks. PaBV-4 RNA in the swabs was quantified by RT-qPCR (**a**) and the time points of first challenge virus detection were plotted (**b**). (**c**) Plasma samples were collected at the indicated time points post PaBV-4 challenge and tested for the presence of PaBV-4-reactive antibodies by iIFT. (**d**) All animals were euthanized at 17 weeks post challenge and organ samples were collected. Viral loads were quantified by RT-qPCR. The position of the X axis indicates the detection limit of the respective test. *P* values < 0.05 indicate significant differences between the groups (Wilcoxon rank sum test).

Starting at six weeks after challenge PaBV-4-specific RNA was detected by RT-qPCR in cloacal swabs of the control group and all birds of this group became virus-positive until week 10 after challenge (Fig. 3a,b), which is in congruence with previous experiences with this virus^17^. Four of the five birds of the vaccine group started shedding viral RNA at 9 to 11 weeks after challenge, while the remaining bird (A6) was tested negative until the end of the experiment at 17 weeks after challenge (Fig. 3a,b). The median onset of viral RNA shedding of the vaccine group was significantly delayed by three weeks as compared to the control group (*P* = 0.0087; Fig. 3b).

Viral distribution in organ samples collected from the control group at 17 weeks after challenge was comparable to previous PaBV-4 infection studies in cockatiels^15^,^17^. Viral RNA levels were lowest in the liver and highest in organs rich of neuronal tissue, such as cerebrum, eye and adrenal gland (Fig. 3d). The overall tissue distribution in four out of five animals of the vaccine group was similar to the control group, but RNA copy numbers were significantly reduced in spleen (*P* = 0.0303) and eye (*P* = 0.0080) as compared to the control group. In congruence with the absence of viral shedding, PaBV-4-specific RNA was not detectable in the organs of the vaccinated animal A6 (Fig. 3d). Titration of infectious virus from brain homogenates of both groups confirmed the results obtained by RT-qPCR (see Supplementary Fig. S4). Further confirmation of the birds´ infection status was obtained by quantification of PaBV-4-reactive serum antibodies at the end of the experiment. All persistently PaBV-4-infected birds of both groups had high antibody titres, which were about ten-fold higher than anti-PaBV-4 titres induced by vaccination. In contrast, serum antibody levels of the vaccinated bird A6 had markedly decreased after challenge infection (Fig. 3c).

### PDD is induced in vaccinated cockatiels after challenge infection with PaBV-4

None of the six cockatiels of the mock-vaccinated group exhibited clinical signs during the experiment or macroscopic lesions at necropsy, although all birds of this group were persistently infected with PaBV-4. In contrast, two of the four persistently infected birds of the vaccine group developed PDD-like disease. Animals A2 and A4 showed mild apathy, ruffled feathers, regurgitation and a transient bodyweight loss of about 15% starting at 9 or 10 weeks after challenge, respectively. In addition, bird A4 started shedding undigested seeds, which continued until the end of the experiment. Both birds slowly regained weight and recovered clinically within the following three to four weeks. At necropsy, the proventriculus of these two birds was moderately dilated (Table 1; see Supplementary Fig. S5).

Microscopic examination revealed mild to severe mononuclear infiltrations in various organs of all PaBV-4-positive birds, but no apparent differences were observed between vaccinated and non-vaccinated birds (Table 1). Birds A2 and A4, which had shown PDD-like clinical signs and gross lesions, were among the birds with the most extended microscopic lesions. Consistent with the lack of challenge virus detection, the vaccinated bird A6 did not show any PDD-related alterations. Overall, most prominent lesions were observed in adrenal gland and proventriculus, whereas mild encephalitis was detectable in only some of the infected birds (Table 1). No lesions were detected in the cerebellum, whereas both groups displayed mononuclear infiltrations in duodenum, kidney, liver and spleen without apparent differences to uninfected controls (see Supplementary Table S1).

### Prime/boost vaccination with NDV and MVA vectors is safe and immunogenic in canaries

**Figure 4:**
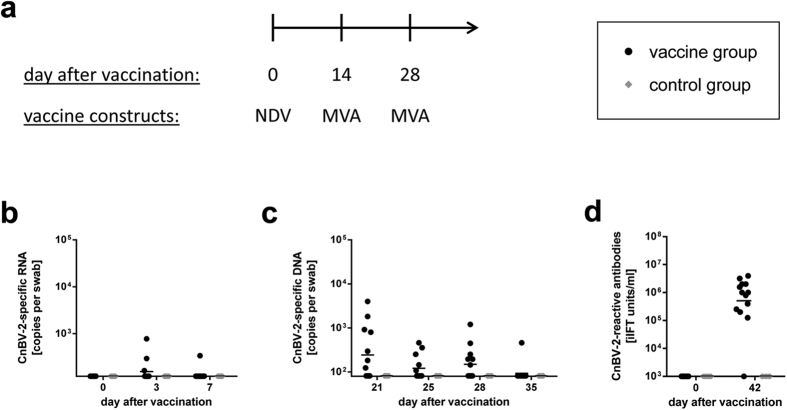
Shedding of vaccine viruses and induction of CnBV-2-reactive antibodies after vaccination of canaries with NDV and MVA vector vaccines (experiment 3). (**a**) A group of 13 canaries (vaccine group) was vaccinated with a mixture of rNDV/CnBV-2/N and rNDV/CnBV-2/P and booster-vaccinated twice with rMVA/CnBV-2/N and rMVA/CnBV-2/P at days 14 and 28 after priming with NDV, while a second group of 13 birds (control group) received the respective parenteral vaccine strains. (**b,c**) Combined pharyngeal and cloacal swabs were collected at the indicated time points. CnBV-2 P RNA originating from rNDV/CnBV-2/P (**b**) or CnBV-2 P DNA from rMVA/CnBV-2/P (**c**) was quantified by qPCR assays. (**d**) Plasma samples were collected at the indicated time points and tested for the presence of CnBV-2-reactive antibodies by iIFT. The positions of the X axes indicate the detection limit of the respective test.

To confirm and extend the results of the cockatiel experiments in a non-psittacine species, an additional vaccination experiment (experiment 3) was performed with common canaries. Two groups of 13 birds each were vaccinated by intramuscular injection with either a mixture of rNDV/CnBV-2/N and rNDV/CnBV-2/P (vaccine group) or with rNDV-wt (control group). Thereafter, both groups were booster-vaccinated twice at intervals of two weeks either with a mixture of rMVA/CnBV-2/N and rMVA/CnBV-2/P or with MVA-wt, respectively (Fig. 4a). In contrast to the experiments with cockatiels, neither infectious NDV nor NDV-specific RNA was detected in swabs collected from NDV-vaccinated canaries (data not shown). However, low levels of CnBV-2 P RNA derived from construct rNDV/CnBV-2/P were detectable in a small number of birds of the vaccine group at days 3 and 7 after vaccination (Fig. 4b). Within the first two weeks after booster vaccination with MVA vaccine viruses, CnBV-2 P DNA derived from rMVA/CnBV-2/P was detected in about half of the swabs collected from the vaccine group, whereas it was barely detectable one week after the second booster vaccination (equalling 21 days after the first booster vaccination; Fig. 4c). Vaccine-derived CnBV-2 P RNA or DNA was not detected in any sample from the control group, confirming that the vectors were not transmitted to this group (Fig. 4b,c).

Similar to cockatiels, none of the vaccinated canaries showed clinical signs following vaccination with NDV or MVA vectors. However, one animal died suddenly during sampling at day 42 after the first vaccination (bird B11; control group). No macroscopic lesions or characteristic microscopic lesions were observed at necropsy.

Serum samples of immunized animals collected at day 42 after vaccination with NDV constructs revealed low levels of NDV-specific HAI antibodies in nine out of 22 tested samples from both groups (see Supplementary Fig. S6), which is comparable to results from vaccinated cockatiels at the same time point (Fig. 2a,b). CnBV-2-reactive antibodies were detectable in all but one animal of the vaccine group, but not in the control group (Fig. 4d), confirming the induction of a bornavirus-specific immune response.

### Vaccinated canaries were partially protected against homologous challenge with CnBV-2 via parenteral routes

**Figure 5:**
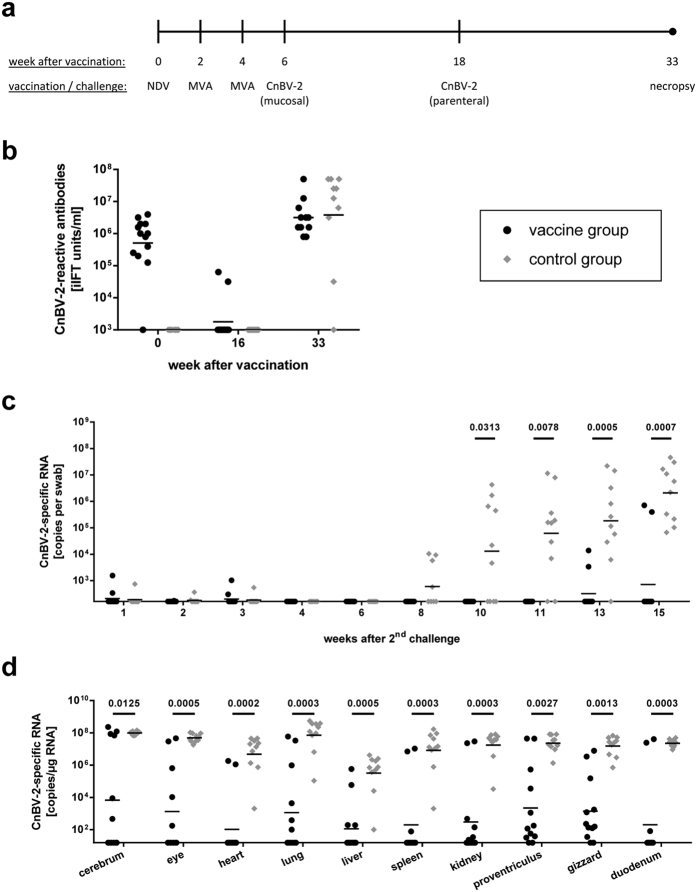
Heterologous prime/boost vaccination against CnBV-2 partially protects canaries against CnBV-2 challenge infection (experiment 3). (**a**) Two groups of 12 or 10 canaries were either vaccinated with a mixture of rNDV/CnBV-2/N and P vectors and boosted twice with rMVA/CnBV-2/N and P (vaccine group) or with the respective parental vaccine viruses (control group). Two weeks after the second booster vaccination, both groups received a homologous challenge infection with CnBV-2 by combined peroral/oculonasal inoculation. Twelve weeks later, the challenge infection was repeated by subcutaneous/intramuscular route. (**b**) Plasma samples were collected at the indicated time points and tested for the presence of CnBV-2-reactive antibodies by iIFT. (**c**) Cloacal swabs were collected at the indicated time points after the 2^nd^ challenge infection and CnBV-2 RNA was quantified by RT-qPCR. (**d**) All animals were euthanized at 15 weeks after the 2^nd^ challenge infection and organ samples were collected. Viral loads were quantified by RT-qPCR. The position of the X axis indicates the detection limit of the respective test. *P* values < 0.05 indicate significant differences between the groups (Wilcoxon rank sum or Wilcoxon signed rank test).

At six weeks after vaccination with NDV constructs (equalling two weeks after the second booster vaccination with MVA viruses), all birds received a high dose of the homologous challenge virus CnBV-2 #15864 (10^5.0^ ffu per bird) by combined peroral and oculonasal inoculation (Fig. 5a). This non-invasive mucosal inoculation route had been proven to successfully establish CnBV-2 infection of canaries in a previous study^5^. However, no evidence of persistent infection was observed for up to 12 weeks after inoculation. Cloacal swabs collected at weekly intervals remained negative for CnBV-2-specific RNA in both groups (data not shown). Furthermore, none of the control-vaccinated animals had seroconverted until 10 weeks after mucosal CnBV-2 inoculation and antibody titres in birds of the vaccine group had considerably decreased as compared to the time point of challenge infection (Fig. 5b). At 11 weeks after mucosal inoculation, two animals of the control group and one animal of the vaccine group were euthanized and organ samples were collected. CnBV-2 RNA was not detected in any organ of the three birds (data not shown), indicating that mucosal infection had failed.

To ensure a reliable challenge infection, all remaining canaries of experiment 3 were inoculated again with isolate CnBV-2 #15864 (10^4.7^ ffu per bird) via parenteral routes (intramuscular and subcutaneous) at 12 weeks after the first challenge infection (equalling 18 weeks after priming with NDV and 14 weeks after the last booster vaccination; Fig. 5a). In congruence with previous findings from experimentally bornavirus-infected canaries^5^,^17^, low amounts of CnBV-2-specific RNA were found in cloacal swabs from a small number of birds during the first three weeks after this challenge infection, but shedding ceased thereafter (Fig. 5c). Beginning in week 8 after challenge, samples collected from the control group again became positive for CnBV-2 RNA and all ten birds of this group started to continuously shed virus until the end of the experiment at 15 weeks after challenge (Fig. 5c). In contrast, only two out of 12 birds of the vaccine group (A1 and A13) were virus-positive in their cloacal swabs, starting in week 11 after challenge, while swabs from the remaining ten birds stayed CnBV-2-negative. Thus, shedding of challenge virus was significantly delayed by vaccination (Fig. 5c).

At necropsy, all birds of the control groups and the two vaccinated animals with CnBV-2-positive swabs (A1 and A13) had the challenge virus widely distributed in all tested organs (Fig. 5d). Two additional birds of the vaccine group contained high viral RNA levels in the brain. Moderate to high viral levels were detected also in the eye of these two birds, but no or only small amounts of virus were present in their peripheral organs. All remaining vaccinated birds showed only low viral levels in one to five organs, particularly in proventriculus and gizzard (Fig. 5d). None of these birds was completely free of detectable CnBV-2 RNA. In congruence with these findings, ten out of 12 vaccinated canaries exhibited high titres of CnBV-2-reactive antibodies in their sera at 15 weeks after challenge (Fig. 5a), indicating a permanent stimulation of their immune system by persisting challenge virus.

None of the CnBV-2-infected canaries in this study developed clinical signs or gross lesions suggestive of PDD. Mild mononuclear infiltrations were observed in the majority of birds, but they were not associated with CnBV-2 infection (see Supplementary Table S2). This is in agreement with previous data from experimental infection of canaries with CnBV-2 or CnBV-1^5^,^17^. One bird of the vaccine group (A6) was found dead during week 10 after challenge without expressing clinical signs prior to death. Macroscopic lesions were not observed during necropsy of this bird but histopathological lesions suggested septicaemia as cause of death, which has not been described as a direct result of bornavirus-induced disease.

## Discussion

The purpose of the present study was to develop viral vector vaccines against avian bornavirus infections which we evaluated for their safety, immunogenicity and protection against homologous bornavirus challenge infection. The predominant target species of our vaccination strategy are Psittaciformes, since bornaviruses are causative agents of the widely distributed and often fatal PDD in this avian order^20^. Therefore, we designed recombinant NDV and MVA vector vaccines carrying the N and P genes of PaBV-4 which is the most widely distributed bornavirus in psittacines^10^,^21^,^51^. This set of vaccines was evaluated in cockatiels, which represent the most commonly used model for bornavirus infections of psittacine birds^14^,^15^,^17^,^18^,^52^,^53^. A second set of NDV and MVA constructs was generated carrying the N and P gene of CnBV-2 to confirm and extend the results in a passerine species, common canary, which is likewise used as an avian bornavirus infection model^5^,^17^.

All vaccine viruses were confirmed to efficiently replicate *in vitro* and to stably express the inserted bornavirus genes over at least five passages in cell culture or embryonated chicken eggs. *In vivo* safety and immunogenicity of the vaccines were evaluated in cockatiels and canaries. A heterologous prime/boost vaccination regime was employed using a mixture of NDV constructs carrying N and P genes for priming, followed by one or two booster vaccinations with the respective mixture of MVA vaccines. The heterologous prime/boost regime was used to prevent vector-directed immunity from interfering with a booster effect against the bornavirus antigens. The experimental design did not allow examination of individual contributions of NDV and MVA vaccines or N and P target genes to immunogenicity and protection. These questions will have to be addressed in subsequent studies.

All vaccine viruses were applied as live vaccines by intramuscular injection. For NDV constructs, application by injection rather than via mucosal surfaces was chosen to reduce the risk of vaccine transmission between the experimental groups, which were housed in separated aviaries within in a single room. The detection of small amounts of infectious NDV and low copy numbers of vaccine-derived nucleic acids on the mucosal surfaces confirmed the NDV and MVA vaccine viruses to initiate a mild self-limiting infection and to spread to the mucosal surfaces in both tested species. Shedding of NDV vaccines was more prominent in cockatiels than in canaries, whereas higher levels of MVA constructs were detected in canaries. Due to the low amounts of infectious virus detected in swab samples and the short duration of vaccine virus shedding, transmission and sustained circulation in vaccinated populations is not expected. The absence of detectable vaccine-derived bornavirus P RNA and DNA in the control groups indicates that the constructs were not transmitted between the experimental groups. Neither cockatiels nor canaries exhibited apparent clinical signs following vaccination with both vectors, further emphasizing the safety of the vaccines.

We assumed that cell-mediated mechanisms rather than humoral immunity were relevant for protection against bornavirus infections^27^,^28^,^29^. However, methods for the detection of specific T lymphocytes are not available for pet bird species such as cockatiels and canaries. Thus, seroconversion had to be used as the sole marker to confirm immunogenicity of the vaccination in both, cockatiels and canaries. In cockatiels, NDV constructs induced moderate levels of vector-specific HAI antibodies, but PaBV-4-reactive antibodies were barely detectable by indirect immunofluorescence test (iIFT) after NDV vaccination. In contrast, high PaBV-4-reactive titres were induced after booster vaccination with MVA constructs. Surprisingly, in experiment 2 an additional booster vaccination of cockatiels with MVA constructs performed two weeks after the first booster vaccination did not result in a detectable increase of antibody titres. It remains unclear whether this was due to immune-mediated restriction of MVA replication. In experiment 3, MVA shedding by vaccinated canaries was markedly reduced after the second MVA vaccination as compared to the first MVA vaccination, likewise indicating immunity against the MVA vector.

To evaluate the protective effect of the vaccination regime against a homologous challenge infection, cockatiels of experiment 1 were inoculated with a high dose of isolate PaBV-4 #6758 (10^4.6^ ffu per bird) by a combined parenteral and mucosal route. While the mock-vaccinated control group developed persistent infection comparable to previous experiments^17^, in the vaccine group shedding of bornaviral RNA was delayed by about three weeks and one out of five vaccinated birds remained completely free of detectable challenge virus. Despite this vaccine-mediated effect, two out of four persistently PaBV-4-infected birds of the vaccine group developed PDD, whereas all six birds of the control group remained clinically healthy but showed microscopic lesions suggestive of PDD. The absence of clinical signs in the control group was rather surprising since in a previous study three out of four cockatiels had developed disease following experimental infection with the same PaBV-4 isolate^17^. However, our results are in agreement with studies of other groups reporting a highly variable morbidity for experimental PaBV-4 infection of cockatiels ranging from 0 to 100%^15^,^18^,^52^,^53^.

Since PDD is discussed to be an immune-mediated disease similar to BD, the disease observed in two of the vaccinated birds may have resulted from vaccine-induced immunopathogenesis. Interestingly, both birds expressed slightly lower viral loads in some of their organs compared to the clinically healthy birds of both groups. Similarly, rats vaccinated with a vacciniavirus vector expressing BoDV-1 N had reduced levels of BoDV-1 in their brains following challenge infection, although they developed more prominent clinical signs and microscopic lesions as compared to non-vaccinated controls^50^. In a further study, adoptive transfer of BoDV-1-specific CD4^+^ T lymphocytes into rats before experimental BoDV-1 infection resulted in elimination of the challenge virus and protected against disease whereas adoptive transfer several days after experimental infection led to a rapid disease induction^29^. This is in line with our hypothesis that an early elimination or at least a permanent restriction of the challenge virus to the site of inoculation are likewise required for clinical protection against PDD, whereas vaccine-induced immunity which is insufficient to prevent viral spread may even exacerbate disease. Notably, the vaccinated bird A6 tested negative for the challenge virus throughout the experiment and did not developed histopathological lesions.

Summing up the results of the first experiment in cockatiels, the vaccine-induced delay of the course of challenge infection demonstrated a promising effect but, unsatisfactorily, the vaccination failed to permanently restrict the challenge infection and did not provide protection against PDD. One reason for non-efficient protection might be the invasive parenteral inoculation with a rather high challenge dose which may have enabled the virus to overcome immunity. Epidemiological data suggests avian bornaviruses are horizontally transmitted^10^ and urofecal-oral transmission is assumed to be a natural infection route^5^,^51^,^54^. However, successful experimental infection of cockatiels via this route has not been reported.

In a previous study we successfully infected a group of seven canaries by combined oculonasal and peroral inoculation with 10^5.4^ ffu of CnBV-2 #15864^5^. Thus, we decided to perform a challenge experiment with canaries, which were inoculated with 10^5.0^ ffu of this virus by a mucosal route. Surprisingly, none of the 25 vaccinated and mock-vaccinated birds showed evidence of persistent CnBV-2 infection for up to 12 weeks thereafter, indicating that the challenge infection had failed. To further investigate this discrepancy, in an additional experiment a group of six non-vaccinated canaries was inoculated with 10^5.2^ ffu of CnBV-2 #15864 by the same route (experiment 4). Only one of the birds unequivocally developed persistent infection as confirmed by seroconversion, shedding of viral RNA and high viral loads widely distributed in its organs (see Supplementary Fig. S7), demonstrating that mucosal inoculation is not a reliable route of CnBV-2 infection of canaries. Potential reasons for the variations between the three experiments are slight differences of the viral dose or immunological status and genetic background of the experimental birds. Furthermore, the presence or absence of mucosal lesions may have influenced the infection efficacy via this route as well as horizontal transmission among experimental birds. In our previous study such lesions may have been induced when collecting pharyngeal swabs shortly before the CnBV-2 inoculation and at weekly intervals thereafter^5^. No such sampling was performed during the corresponding periods of experiments 3 and 4 described here.

As a consequence of the unsuccessful mucosal challenge infection, a second challenge infection was performed by parenteral routes at 14 weeks after the last booster vaccination. This time all animals of the control group developed persistent infection with viral shedding and high viral loads detectable in all tested organs. In contrast, the course of infection was dramatically delayed in the vaccine group with only two out of 12 birds exhibiting viral shedding at the end of the experiment at week 15 after challenge. The majority of vaccinated birds had only low levels of viral RNA detectable in individual organs but sterile immunity was not achieved. It remains unknown whether some or all of these birds would have started to shed virus at later time points or whether vaccine-induced immunity was able to permanently block virus distribution in their organisms. Neither vaccinated nor non-vaccinated canaries developed PDD-like disease or overt microscopic lesions after challenge infection, which is in agreement with previous experimental bornavirus infections of this species^5^,^17^.

In summary, this study reports the first promising immunoprophylaxis strategy against avian bornavirus infections. The use of a heterologous prime/boost regime employing NDV and MVA markedly delayed the course of challenge infections in two different avian species. However, vaccine-induced immunity was not able to prevent PDD and related microscopic lesions in PaBV-4-infected cockatiels, suggesting that sterile immunity or at least permanent control of the virus at a very early stage of infection may be required for clinical protection. It may be speculated that the high dose and invasive route of experimental challenge infection may not be representative for natural bornavirus infection, possibly leading to an underestimation of the protective effect. Therefore, further research should focus not only on the immune mechanisms involved in protection and disease induction, but also on investigating natural routes of avian bornavirus transmission.

## Material and Methods

### Avian bornaviruses and virus stock preparation

PaBV-4 #6758 (GenBank accession number FJ603685) was isolated from a blue-and-yellow macaw (*Ara ararauna*) suffering from PDD^51^,^55^. In a previous study we could demonstrate this virus to induce PDD in experimentally infected cockatiels^17^. CnBV-2 #15864 (KC464478) was isolated from a common canary exhibiting a dilated proventriculus. Experimental inoculation of canaries with this virus via parenteral and mucosal routes established persistent infection but did not result in clinical disease^5^. Isolation of PaBV-4 #6758 and CnBV-2 #15864 and preparation of virus stocks from persistently infected CEC-32 quail fibroblast cells or QM7 quail smooth muscle cells, respectively, were described previously^5^,^17^,^51^.

### Generation of recombinant MVA and NDV vectors carrying N and P genes of PaBV-4 and CnBV-2

Recombinant MVA vaccine viruses carrying the N or P gene of PaBV-4 #6758 (designated rMVA/PaBV-4/N and rMVA/PaBV-4/P) or CnBV-2 #15864 (rMVA/CnBV-2/N and rMVA/CnBV-2/P) were generated by homologous recombination following previously described procedures^41^. Briefly, cells infected with MVA strain F6 (designated MVA-wt; kindly provided by Gerd Sutter, Munich, Germany) were transfected with a pIIIH5redK1L transfer plasmid carrying the corresponding bornavirus open reading frame (ORF) and recombinant viruses were selected with the help of K1L and mCherry marker genes during repeated passaging on rabbit RK13 cells and CEF cells. For the #15864 N gene, a poxvirus transcription stop signal (TTTTTAT) starting at nucleotide position 918 had been changed to TTTCTAT by introducing a synonymous T to C mutation at position 921 using QuikChange Site-directed mutagenesis kit (Agilent Technologies).

A set of recombinant NDV vaccine viruses carrying the same bornavirus genes (designated rNDV/PaBV-4/N, rNDV/PaBV-4/N, rNDV/CnBV-2/N, rNDV/CnBV-2/P) was constructed applying a previously published reverse genetic system for the lentogenic NDV vaccine strain Clone 30^40^,^56^. Plasmids encoding the full-length NDV genome with a bornavirus ORF inserted between the NDV fusion protein and hemagglutinin-neuraminidase genes were used for virus rescue from transfected BSR-T7 cells^56^,^57^.

Stocks of rMVA and rNDV viruses were generated in CEF cultures or embryonated chicken eggs, respectively, following previously described procedures^40^,^41^.

The correct insertion of bornavirus genes was confirmed by RT-PCR of selected genome regions and subsequent sequencing. The expression of bornavirus N or P proteins was demonstrated by immunofluorescence staining (see Supplementary Fig. S1). To demonstrate their genetic stability, rNDV and rMVA viruses were propagated for five passages in either embryonated chicken eggs or CEF cultures, respectively (see Supplementary Fig. S2). All recombinant viruses grew to comparable titres on CEF cells, and MVA constructs were confirmed not to replicate in human HeLa cells (data not shown).

### Experimental animals

Twenty-four cockatiels and 32 canaries were included in this study. Cockatiels originated from the breeding flocks of scientific institutions in Germany which were regularly monitored for the presence of psittacine pathogens including avian bornaviruses. Canaries of the breeds “intensive red” and “lizard” were derived from canary breeders whose flocks had been tested negative for avian bornaviruses. All birds were clinically healthy and neither avian bornavirus RNA nor bornavirus-reactive antibodies were detected in cloacal swabs or serum samples, respectively, collected prior to the experiments. Birds were housed in an aviary under biosecurity level (BSL) 2 conditions and provided with commercial conure or canary feed, fruits and water. Housing conditions were described in more detail previously^17^.

Animal experiments were performed in compliance with the German animal protection law (TierSchG). The animals were housed and handled in accordance with good animal practice as defined by FELASA (http://www.felasa.eu/recommendations) and the national animal welfare body GV-SOLAS (http://www.gv-solas.de). All animal experiments were approved by the animal welfare committees of the local authorities (Regierungspräsidium Freiburg; application number 35–9185.81/G-13/55).

### Experimental infection of vaccinated cockatiels and canaries

Safety and immunogenicity of NDV and MVA vaccines administered to cockatiels or canaries in a heterologous prime/boost regime was tested in three independent experiments. Furthermore, in two of these experiments the protective effect against challenge infection with a homologous bornavirus was investigated.

In the first experiment (experiment 1), twelve cockatiels aged four to six months were divided into two groups of six birds each. One group (vaccine group) received a mixture of equal amounts of rNDV/PaBV-4/N and rNDV/PaBV-4/P (10^5.9^ ffu of each virus per bird) by intramuscular injection. The second group (control group) was injected with an equivalent dose of the rNDV vector not expressing foreign genes (designated rNDV-wt; 10^6.2^ ffu per bird). Three weeks after vaccination both groups received a heterologous booster vaccination with either a mixture of rMVA/PaBV-4/N and rMVA/PaBV-4/P (10^7.7^ ffu of each virus per bird; vaccine group) or MVA-wt (10^8.0^ ffu per bird; control group) by intramuscular injection. At three weeks after booster vaccination homologous challenge infection was performed with isolate PaBV-4 #6758 (10^4.6^ ffu per bird) by combined intramuscular, subcutaneous, peroral and oculonasal application. The experiment was terminated 17 weeks after challenge and all birds were euthanized.

A second experiment (experiment 2) was performed with twelve approximately three-year-old cockatiels, which were divided into two groups of six birds each. Similar to the first experiment, the vaccine group was first vaccinated with a mixture of rNDV/PaBV-4/N and rNDV/PaBV-4/P (10^6.1^ ffu per virus and bird), but in this experiment it was followed by two booster vaccinations with rMVA/PaBV-4/N and rMVA/PaBV-4/P (10^7.7^ ffu of each virus per bird and injection) at 14 and 29 days after the first vaccination. In parallel, the control group was vaccinated with equivalent doses of the rNDV-wt and MVA-wt.

In a third experiment (experiment 3), 26 juvenile canaries (breed “intensive red”; age two to four months) were divided into two groups of 13 birds. The vaccine group was vaccinated with a mixture of rNDV/CnBV-2/N and rNDV/CnBV-2/P (10^6.6^ ffu per virus and bird), followed by two booster vaccinations at days 14 and 28 after the first vaccination with mixtures of rMVA/CnBV-2/N and rMVA/CnBV-2/P (10^7.4^ ffu of each virus per bird and injection), while the control group received equivalent doses of rNDV-wt and MVA-wt. Six weeks after the first vaccination (equalling two weeks after the last booster vaccination) both groups received a homologous challenge infection with CnBV-2 #15864 (10^5.0^ ffu per bird) by combined peroral and oculonasal inoculation. Twelve weeks later the challenge infection was repeated by combined intramuscular and subcutaneous inoculation (10^4.7^ ffu per bird). The experiment was terminated at 15 weeks after the second challenge infection.

In a further experiment (experiment 4), six non-vaccinated juvenile canaries (breed “lizard”) were inoculated with CnBV-2 #15864 (10^5.2^ ffu per bird) via combined peroral and oculonasal route. The inoculation was performed in parallel with the second challenge infection of experiment 3 with the same virus preparation. Birds of both experiments were housed together in the same aviary.

In all experiments, the vaccine and control groups were housed in two separate aviaries located in the same room (distance 1.5 m). After challenge infection the aviaries were combined and both groups were housed together. Animals were observed daily for the presence of clinical signs and body weights were determined at weekly intervals. Combined pharyngeal and cloacal swab samples or cloacal swabs were collected for detection of vaccine and challenge viruses by virus titration or PCR assays and serum samples were collected for detection of NDV- and bornavirus-specific antibodies. At the end of each experiment, euthanized birds were necropsied and organ samples were collected for virus detection and histopathological analysis.

### Detection of bornavirus RNA by RT-qPCR assays

Bornavirus- and NDV-derived RNA and MVA-derived DNA from virus-infected cell cultures as well as from combined pharyngeal and cloacal swabs collected during the vaccination experiments were extracted using QIAamp viral RNA mini kit (Qiagen). RNA extraction from homogenized organ samples was performed by phenol-chloroform extraction with Trifast (Peqlab). For detection of viral RNA, reverse transcription (RT) was performed with Revertaid reverse transcription reagents (Thermo Scientific). Detailed procedures have been described previously^5^,^17^,^51^.

Two qPCR assays targeting the P genes of either PaBV-4^7^,^17^ or CnBV-2 (see Supplementary Table S3) were applied for detection of challenge virus and NDV and MVA vaccine viruses carrying bornavirus P genes. Dilution series of plasmids containing the complete P gene of either PaBV-4 #6758 or CnBV-2 #15864 were used as standard curves for the estimation of copy numbers. Detailed procedures have been described elsewhere^17^.

### Virus titration

Shedding of infectious vaccine virus was quantified by virus titration from freshly collected combined pharyngeal and cloacal swabs. MVA titration was performed on chicken DF-1 cells whereas NDV was titrated in MDCK cultures. Cells were incubated for 72 h before virus-positive cell foci were visualized by immunoperoxidase staining with TrueBlue substrate (KPL) using either polyclonal rabbit-anti-NDV or rabbit-anti-VACV/A27L as detection serum. Viral titres were calculated as ffu/ml. Titres of vaccine stocks were determined by the same method.

### Detection of NDV- and bornavirus-specific antibodies

NDV-specific antibodies in cockatiel and canary sera were quantified by HAI test with pre-adsorption of the sera following previously published procedures^58^. Antibody titres were recorded as the reciprocal serum dilution able to completely inhibit hemagglutination.

The presence of bornavirus-specific antibodies was measured by iIFT following procedures published in detail elsewhere^17^,^48^. For detection of PaBV-4-reactive antibodies in cockatiel sera, QM7 cells persistently infected with PaBV-4 #6758 were used as target cells, whereas Vero cells persistently infected with CnBV-2 #15864 were used for the analysis of canary sera. Titres were calculated as endpoint titres per ml serum.

### Histopathological analysis

Tissue slices from experimental animals were stained with haematoxylin and eosin (HE) and analysed in parallel with tissues from three uninfected cockatiels or four uninfected canaries originating from the same flocks. Histopathological analysis was performed independently by two experienced pathologists (C.H., S.M.), who were blinded to the identity of the samples. Scores were assigned as “−” (no or only physiological levels of mononuclear cells present in the tissue), “+”, “++” or “+++” (mild, moderate or severe mononuclear infiltration). Alterations other than inflammatory lesions were described qualitatively.

### Statistical analysis

Viral quantities and beginning of challenge virus shedding were compared between vaccine and control groups by two-tailed Wilcoxon rank sum and Wilcoxon signed rank test using GraphPad Prism 6 software. *P* values lower than 0.05 were considered to indicate significant differences.

## Additional Information

**How to cite this article**: Olbert, M. *et al*. Viral vector vaccines expressing nucleoprotein and phosphoprotein genes of avian bornaviruses ameliorate homologous challenge infections in cockatiels and common canaries. *Sci. Rep*. **6**, 36840; doi: 10.1038/srep36840 (2016).

**Publisher's note:** Springer Nature remains neutral with regard to jurisdictional claims in published maps and institutional affiliations.

## Supplementary Material

Supplementary Information

## Figures and Tables

**Table 1:** Clinical signs, macroscopic and microscopic lesions observed after PaBV-4 challenge infection of cockatiels (experiment 1).

Group Bird	Clinical disease^a^	Proven tricular dilatation	Mononuclear infiltration score						
			cere-brum	adrenal gland	heart	pan-creas	lung	proven-triculus	gizzard
vaccine group									
A1	−	−	−	++	++	+	−	−	−
A2	+	+	++	n.a.^b^	+++	++	+	++	++
A4	+	+	+	+++	+++	−	+	+++	+++
A5	−	−	+	+++	++	++	−	++	−
A6	−	−	−	−	−	−	−	−	−
control group									
B1	−	−	+	+++	−	++	−	++	++
B2	−	−	++	+++	+++	+	−	++	++
B3	−	−	−	+++	+	−	++	++	−
B4	−	−	−	++	−	+++	−	++	−
B5	−	−	−	++	++	−	−	++	+
B6	−	−	+	n.a.	+	−	+	++	+
uninfected group^c^									
C1	−	−	−	−	−	−	−	+	−
C2	−	−	−	−	−	n.a.	−	−	−
C3	−	−	−	−	−	−	−	−	−

^a^Clinical signs included shedding of undigested seeds, regurgitation, ruffled feathers and transient weight loss.

^b^n.a. =  not analyzed.

^c^Uninfected cockatiels originating from the same flock as the experimental birds served as controls.
