# Supplementary material for: Viral vector vaccines expressing nucleoprotein and phosphoprotein genes of avian bornaviruses ameliorate homologous challenge infections in cockatiels and common canaries
Source: Sci Rep. 2016 Nov 10;6:36840. doi: 10.1038/srep36840 (PMC5103271; doi:10.1038/srep36840)
Supplement: Supplementary Information [file srep36840-s1.pdf]

SUPPLEMENTARY INFORMATION

Viral vector vaccines expressing nucleoprotein and phosphoprotein genes of avian bornaviruses ameliorate homologous challenge infections in cockatiels and common canaries

Marita Olbert, Angela Römer-Oberdörfer, Christiane Herden, Sara Malberg, Solveig Runge, Peter Staeheli and Dennis Rubbenstroth

Supplementary Fig. S1. Recombinant NDV and MVA vaccine viruses express bornavirus N and P proteins in chicken embryo fibroblast (CEF) cultures. CEF cultures were infected with rNDV (A) or rMVA (B) vectors at an MOI of 0.01 or 0.002, respectively. After incubation for 24 or 48 h, respectively, immunofluorescence staining of parallel wells was performed with one of the following polyclonal sera: rabbit-anti-PaBV-2/N[1], rabbit-anti-BoDV-1/P[2], rabbit-anti-NDV or rabbit-anti-VACV/A27L (Acris Antibodies, Herford, Germany) following previously published procedures[2]. QM7 cells persistently infected with PaBV-4 or CnBV-2 were used as positive controls.

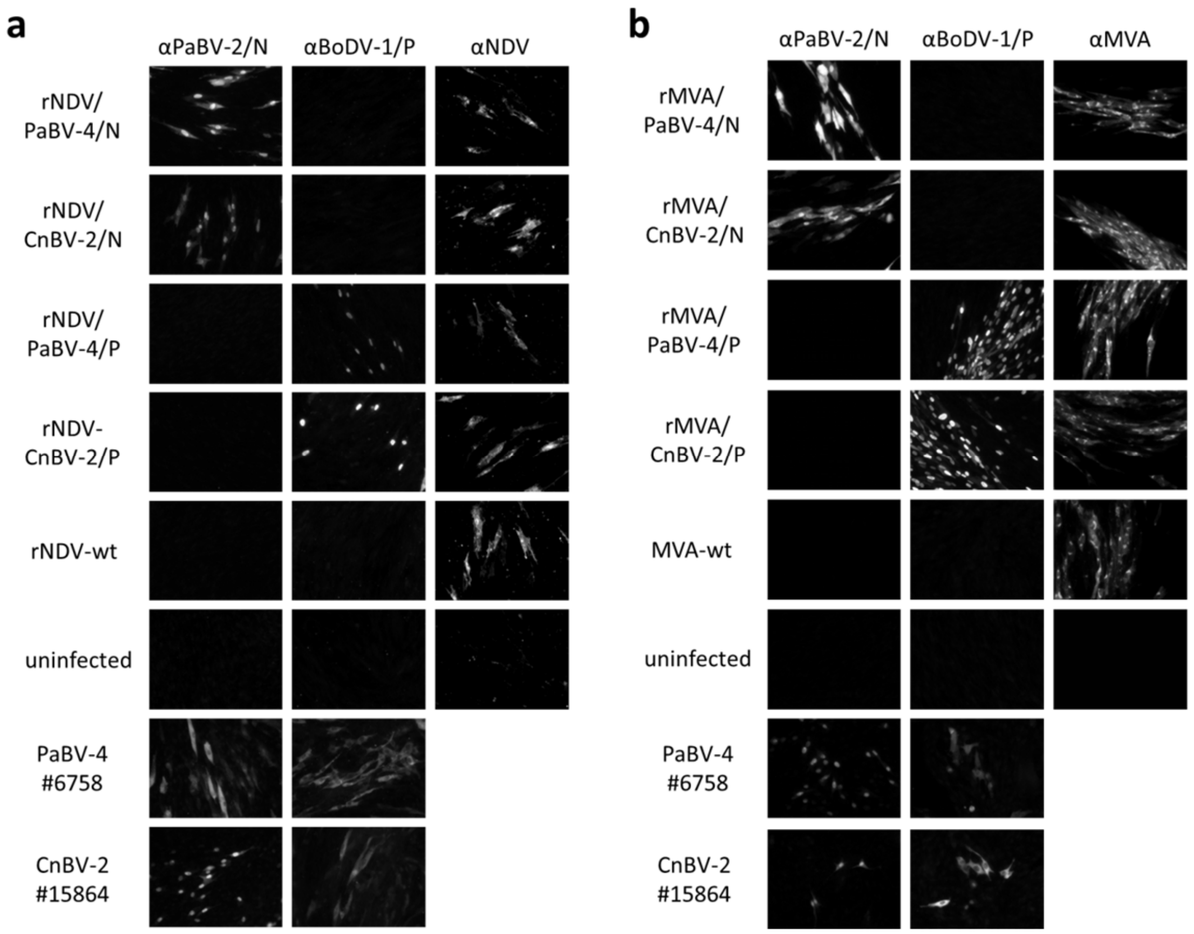

Supplementary Fig. S2. Bornavirus protein expression is retained after passaging of vaccine viruses in culture systems. rNDV (A) and rMVA (B) constructs were passaged five times in either CEF cultures or embryonated chicken eggs, respectively. Following the fifth passage, CEF cultures were infected and bornavirus N or P protein expression was visualized by immunofluorescence staining with the indicated antibodies as described for Supplementary figure S1.

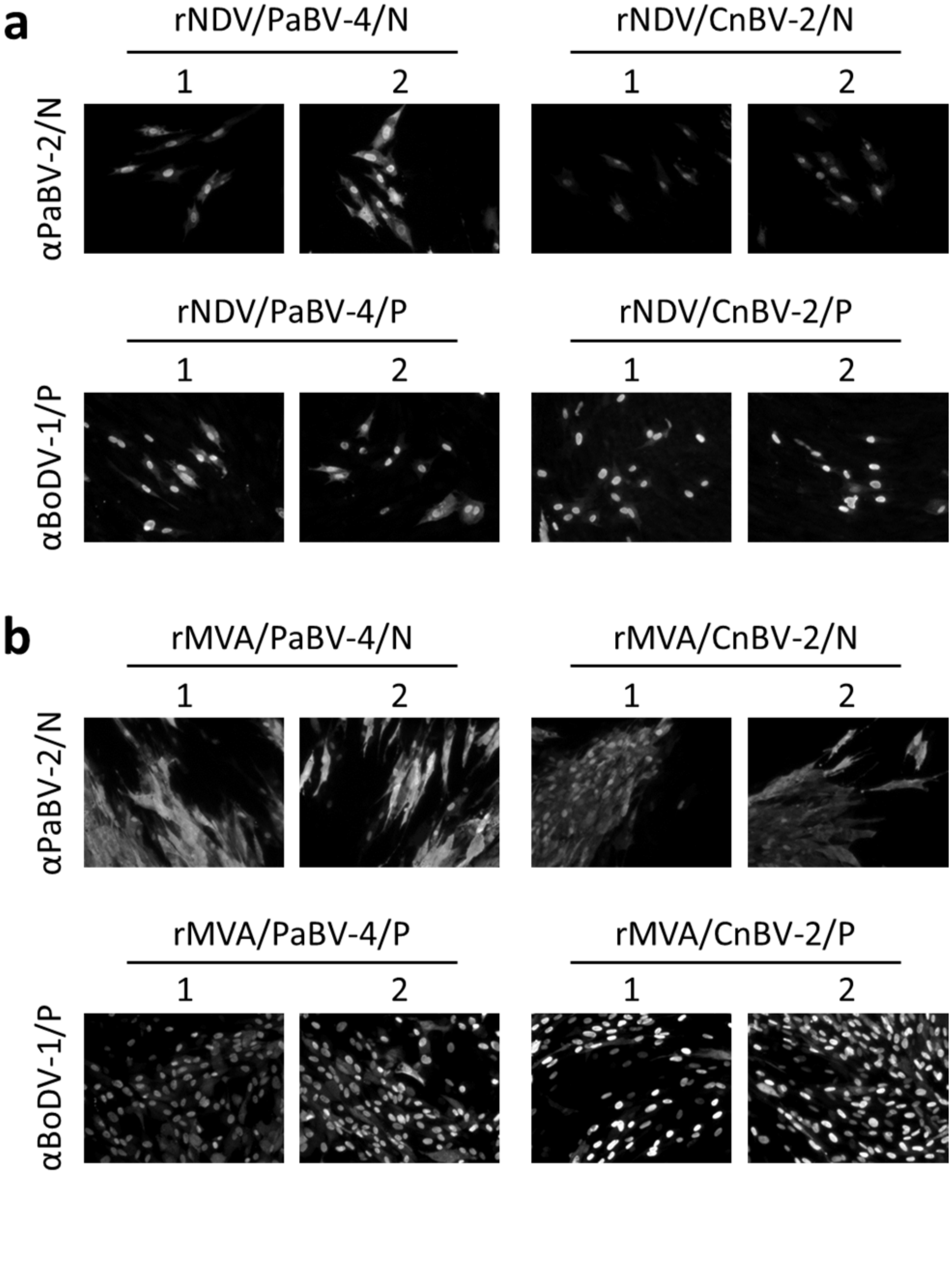

Supplementary Fig. S3. Shedding of NDV and MVA vaccine viruses after vaccination of cockatiels (experiments 1 & 2). (A) During two experiments groups of six cockatiels were either vaccinated with a mixture of rNDV/PaBV-4/N and rNDV/PaBV-4/P and subsequently booster-vaccinated with rMVA/PaBV-4/N and rMVA/PaBV-4/P (vaccine groups) or received the parenteral strains rNDV-wt and MVA-wt (control group). Combined pharyngeal and cloacal swabs were collected at the indicated time points. (B, C) NDV was detected by conventional RT-PCR[3]. (D) Vaccine-derived PaBV-4 P DNA originating from rMVA/PaBV-4/P was quantified by qPCR. In experiment 1 no PaBV-4 P DNA was detectable (data not shown). The position of the X axis indicates the detection limits of the test.

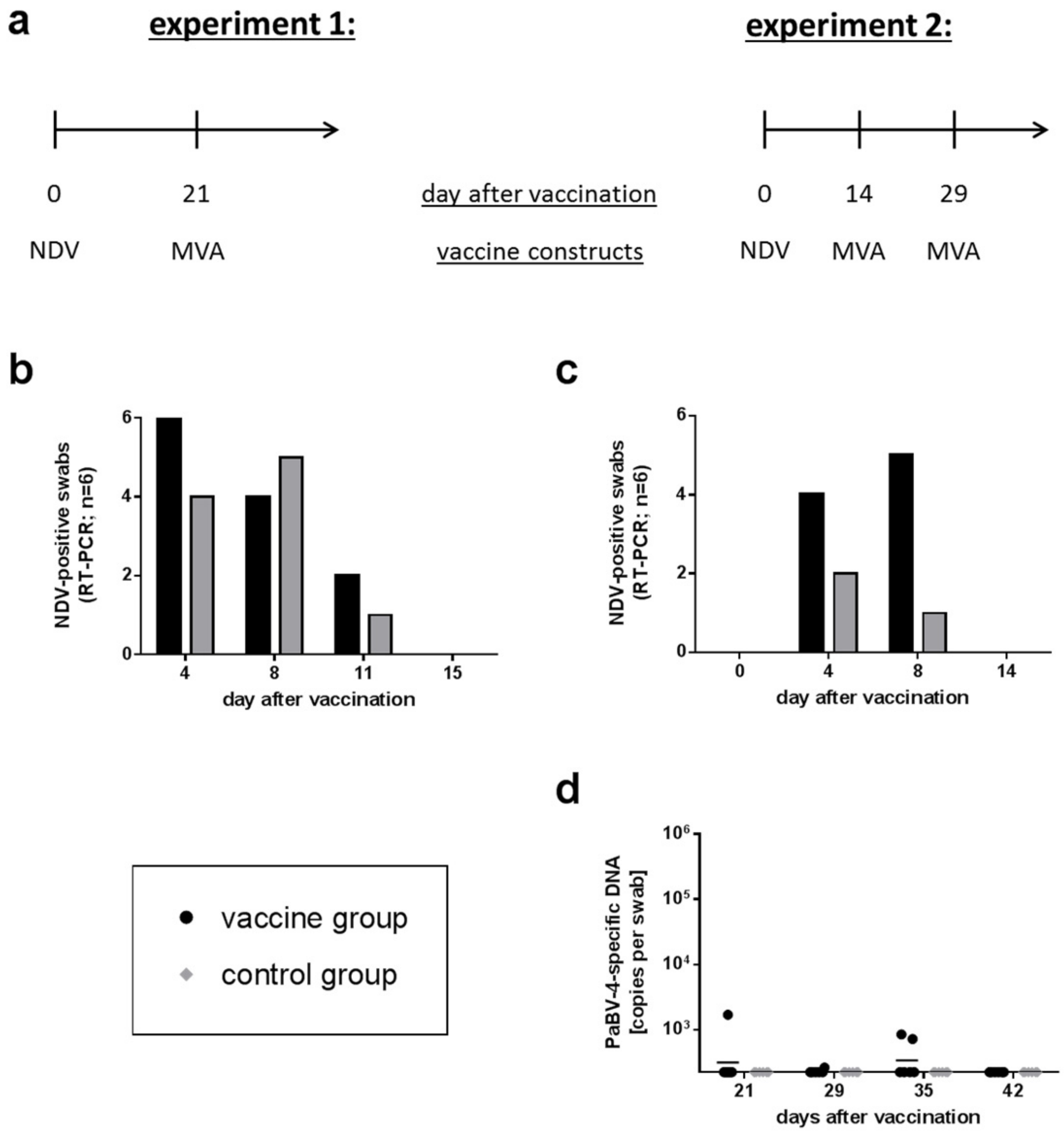

Supplementary Fig. S4. Infectious bornavirus titers in the brains of cockatiels and canaries after challenge infection (experiments 1 & 3). Brain samples were collected during necropsy at 17 or 15 weeks after challenge of cockatiels with PaBV-4 (A; experiment 1) or canaries with CnBV-2 (B; experiment 3), respectively. Ten-fold dilution series of ultrasonicated tissue homogenates were incubated with CEC-32 quail fibroblasts (A) or QM7 quail muscle cells (B) for four days before virus-positive cell foci were visualized by immunofluorescence staining and foci-forming units (ffu) were calculated. The procedure has been described in more detail elsewhere[4-6]. The position of the X axis indicates the detection limit of the test. *P* values < 0.05 indicate significant differences between the groups (Wilcoxon rank sum test).

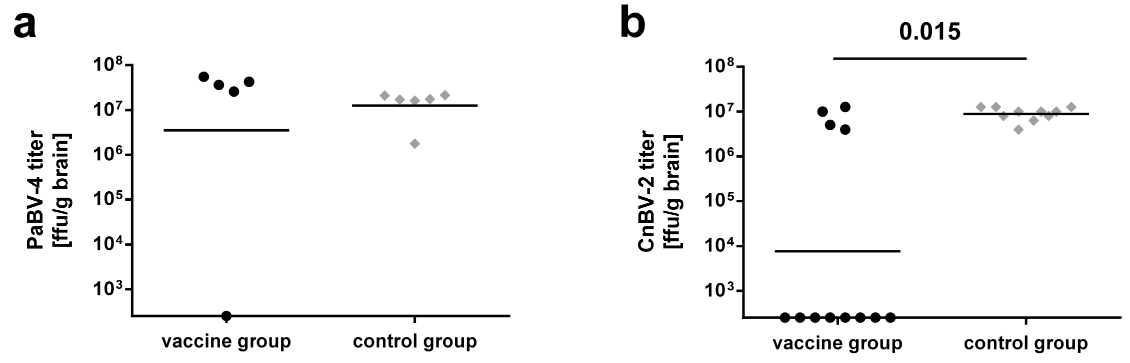

Supplementary Fig. S5. Persistently PaBV-4-infected cockatiels develop proventricular dilatation despite vaccination with NDV and MVA vector vaccines expressing bornavirus antigens (experiment 1). Two groups of cockatiels were either vaccinated with a mixture of rNDV/PaBV-4/N and rNDV/PaBV-4/P and booster-vaccinated with rMVA/PaBV-4/N and rMVA/PaBV-4/P (vaccine group) or received the parenteral strains rNDV-wt and MVA-wt (control group). Subsequently, both groups received a homologous challenge infection with PaBV-4 #6758. Seventeen weeks after challenge infection all birds were euthanized and necropsied. Bird A6 (red box) remained free of detectable challenge virus whereas all other birds were persistently infected. Birds A2 and A4 (blue boxes) of the vaccine group showed a moderate dilatation of the proventriculus.

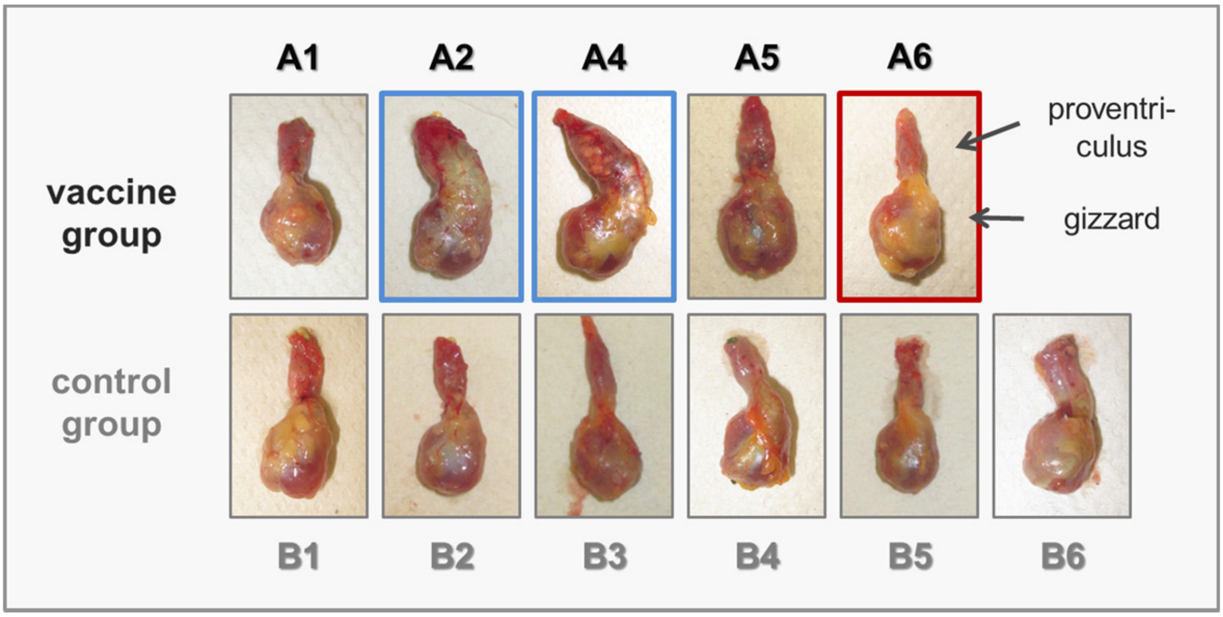

Supplementary Table S1. Microscopic lesions observed after PaBV-4 challenge infection of cockatiels (experiment 1).

Group	Mononuclear infiltration score				
Bird	cerebellum	spleen	liver	kidney	duodenum
vaccine group					
A1	-	+	+	++	++
A2	-	++	++	+	++
A4	-	+++	-	+	++
A5	-	+	+	++	++
A6	-	++	++	-	+++
control group					
B1	-	-	+++	+++	++
B2	-	+++	+	+++	+++
B3	-	++	+	++	++
B4	-	+++	+	++	++
B5	-	++	++	++	++
B6	-	++	++	+++	++
uninfected group [a]					
C1	-	+++	++	+	+++
C2	-	+++	-	++	++
C3	-	+++	++	+	++

[a] Uninfected cockatiels originating from the same flock as the experimental birds served as controls.

Supplementary Fig. S6. Detection of NDV-specific HAI antibodies in vaccinated canaries (experiment 3). Two groups of 13 canaries each were vaccinated either with a mixture of rNDV/CnBV-2/N and rNDV/CnBV-2/P (vaccine group) or with the parental strain rNDV-wt (control group). Plasma samples were collected at the indicated time points after vaccination and tested for the presence of NDV-specific antibodies by HAI test. Due to limited sample volumes not all birds were tested at both time points. The position of the X axis indicates the detection limit of the test.

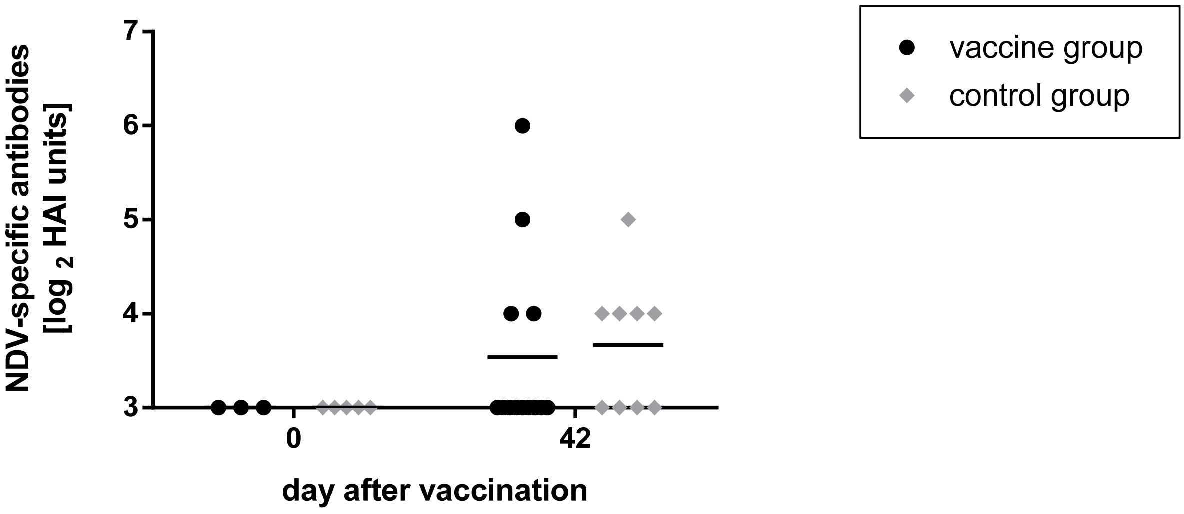

Supplementary Table S2. Microscopic lesions in tissues of CnBV-2-infected canaries (experiments 3 & 4).

Group<br>Bird	CnBV-2-pos. organs (RT-qPCR; n=10)	Mononuclear infiltration score<br>cere-brum	 cere-bellum	 heart	 pan-creas	 proven-triculus	 gizzard	 duode-num	 liver
experiment 3 – vaccine group [a]									
A1	10	-	-	++	-	++	-	-	++
A2	1	-	+	-	n.a. [e]	-	-	n.a.	++
A3	1	+	-	++	n.a.	-	-	n.a.	++
A4	2	-	-	++	+	+	+	-	+++
A5	5	-	-	-	-	-	-	-	++
A6 [b]	5	-	n.a.	++	n.a.	+	++	-	-
A7	4	-	n.a.	+	-	+	+	-	+
A8	5	+++	-	-	-	+	-	-	+
A9	2	+	-	++	++	++	-	++	+++
A11	7	-	-	+	-	-	-	-	+++
A12	3	+	+	+	-	n.a.	-	+	+
A13	10	-	-	-	-	++	-	++	++
experiment 3 – control group [a]									
B2	10	-	+	+++	+	+	+	+	++
B3	10	-	-	+	-	+	-	-	++
B4	10	-	-	+	-	-	-	++	++
B5	10	-	-	++	-	++	+	++	++
B6	10	+	-	+	+	+	-	+	++
B7	10	-	-	+	-	n.a.	-	n.a.	++
B9	10	-	-	++	n.a.	++	-	+	+
B10	10	++	+	++	++	-	-	+	+++
B12	10	++	-	+	-	+	+	-	+++
B13	10	++	+	+	-	++	-	-	+
uninfected birds [c]									
C1	-	-	-	+	n.a.	n.a.	-	-	+++
C2	-	+	-	+	n.a.	+	-	+	+
C3	-	-	-	+	n.a.	-	+	-	+
C4	-	-	-	+	n.a.	-	-	+	++
experiment 4 [d]									
D1	1	-	-	-	-	+	-	+	++
D2	-	-	-	+	n.a.	+++	-	++	+++
D3	1	-	-	++	-	++	-	++	+++
D4	-	-	-	+	n.a.	+	+	-	+++
D5	10	-	-	+	n.a.	+	+	+	+++
D6	2	-	-	+	-	-	+	+	+++

[a] Birds vaccinated with rNDV and rMVA viruses expressing bornavirus proteins (vaccine group) or with rNDV-wt and MVA-wt (control group) were challenged with CnBV-2 #15864 by parenteral injection and necropsied at 15 weeks after challenge. Birds A10, B1, B8 and B11 had died or been euthanized prior to parental challenge.

[b] Animal A6 was found dead during week 10 after parental challenge infection.

[c] Uninfected birds originated from the same flock as experimental birds of experiment 3.

[d] Non-vaccinated birds were inoculated with CnBV-2 #15864 via mucosal routes and necropsied at 15 weeks post infection.

[e] n.a. = not analyzed

Supplementary Fig. S7. CnBV-2 does not reliable establish persistent infection in canaries when administered via mucosal surfaces (experiment 4). Six juvenile non-vaccinated, bornavirus-free canaries (breed "lizard") were inoculated with CnBV-2 #15864 ($10^{5.2}$ ffu per bird) by combined peroral and oculonasal route. (A) Plasma samples were collected at the indicated time points and tested for the presence of CnBV-2-reactive antibodies by iIFT. (B) Cloacal swabs were collected at intervals of one to two weeks and CnBV-2 RNA was quantified by RT-qPCR (C) All animals were euthanized at 15 weeks post infection and organ samples were collected. Viral loads were quantified by RT-qPCR. The position of the X axis indicates the detection limit of the respective test.

Only one of the six canaries (bird D5) unequivocally developed persistent infection as confirmed by seroconversion (A), shedding of viral RNA (B), and high viral loads widely distributed in organ samples (C). For three additional animals, very low amounts of viral RNA were detectable in heart or eye (C). It remains questionable, whether the virus detected in the eyes of these three birds reflects a persistent infection which is still largely restricted to the site of inoculation. Another possible explanation is a contamination of the ocular surface with dust containing sheddings of CnBV-2-infected birds housed in the same aviary.

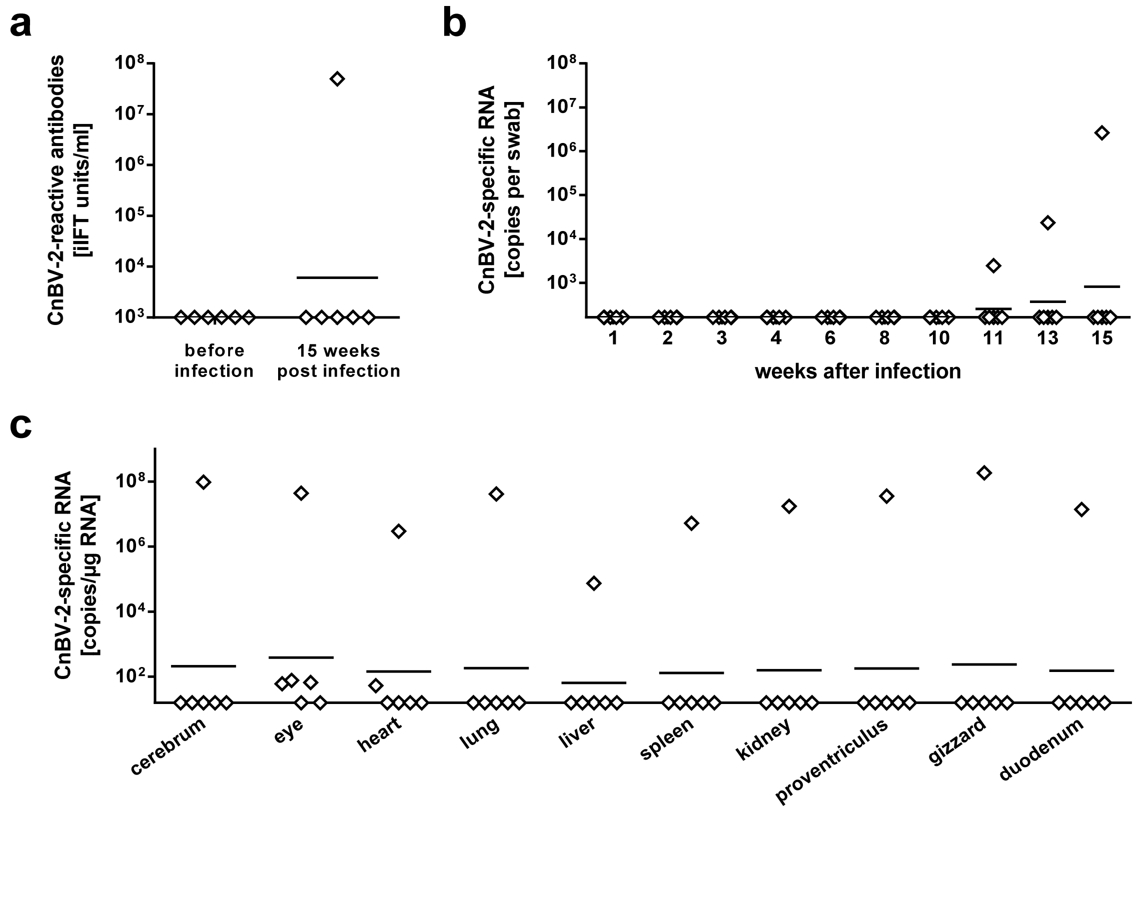

Supplementary table S3. Primers and TaqMan probes used in this study.

Target	Primer/probe	Sequence (5' to 3')	Reference
NDV	PMV-NP-F2	AGGCGCAAAGCTCATCTG	Barbezange & Jestin[3]
	PMV-NP-R2	TTGCCACTGCTCTCATCA	
PaBV-4 P gene	1034/1322-F	CAGACAGCACGTCGAGTGAGA	Honkavuori et al.[7]
	1034/1322-R	AGTTAGGGCCTCCCTGGGTAT	
	1034/1322-P	6FAM-AGGTCCCCGCGAAGGAAGCGA-BHQ1	
CnBV-2 P gene	CnBV-2_1392+	CCAGCCGGTAGAGCATCTTC	this study
	CnBV-2_1484-	TTCGACAACTGCTCCCTTCC	
	CnBV-2_1430_P	6FAM-ACCCATCCATGATCTCCGACCCAGACC-BHQ1	

References

1 Reuter, A. *et al.* Avian Bornaviruses escape recognition by the innate immune system. *Viruses* **2**, 927-938 (2010).

2 Zimmermann, V., Rinder, M., Kaspers, B., Staeheli, P. & Rubbenstroth, D. Impact of antigenic diversity on laboratory diagnosis of Avian bornavirus infections in birds. *J Vet Diagn Invest* **26**, 769-777, doi:10.1177/1040638714547258 (2014).

3 Barbezange, C. & Jestin, V. Development of a RT-nested PCR test detecting pigeon Paramyxovirus-1 directly from organs of infected animals. *J Virol Methods* **106**, 197-207 (2002).

4 Rubbenstroth, D. *et al.* No contact transmission of avian bornavirus in experimentally infected cockatiels (*Nymphicus hollandicus*) and domestic canaries (*Serinus canaria* forma domestica). *Vet Microbiol* **172**, 146-156, doi:10.1016/j.vetmic.2014.05.011 (2014).

5 Rubbenstroth, D., Rinder, M., Kaspers, B. & Staeheli, P. Efficient isolation of avian bornaviruses (ABV) from naturally infected psittacine birds and identification of a new ABV genotype from a salmon-crested cockatoo (*Cacatua moluccensis*). *Vet Microbiol* **161**, 36-42 (2012).

6 Rubbenstroth, D. *et al.* Avian bornaviruses are widely distributed in canary birds (*Serinus canaria* f. domestica). *Vet Microbiol* **165**, 287-295, doi:10.1016/j.vetmic.2013.03.024 (2013).

7 Honkavuori, K. S. *et al.* Novel borna virus in psittacine birds with proventricular dilatation disease. *Emerg Infect Dis* **14**, 1883-1886 (2008).
